# Single-Cell Analysis Reveals Isotype-Specific Autoreactive B Cell Repertoires in Sjögren’s Syndrome

**DOI:** 10.1371/journal.pone.0058127

**Published:** 2013-03-13

**Authors:** Cuong Q. Nguyen, Adebola O. Ogunniyi, Afife Karabiyik, J. Christopher Love

**Affiliations:** 1 Department of Infectious Diseases and Pathology, College of Veterinary Medicine, University of Florida, Gainesville, Florida, United States of America; 2 Center for Orphan Autoimmune Disorders, College of Dentistry, University of Florida, Gainesville, Florida, United States of America; 3 Department of Chemical Engineering, Massachusetts Institute of Technology, Cambridge, Massachusetts, United States of America; 4 Koch Institute for Integrative Cancer Research, Cambridge, Massachusetts, United States of America; National Institute of Dental and Craniofacial Research, United States of America

## Abstract

Microengraving is a novel technology that uses an array of microfabricated subnanoliter wells to isolate and characterize secreted proteins from larger number of single cells. This printing technique permits the capture and characterization of secreted antibodies on glass slides. Here, we profiled the antigenic repertoires of B cells reacting against salivary gland tissues in Sjögren’s syndrome (SjS), an autoimmune disease targeting the exocrine glands. Single-cell suspensions of spleen and cervical lymph node cells prepared from normal C57BL/6 and SjS-susceptible (SjS^s^) C57BL/6.NOD-*AecAec2* mice were dispersed into subnanoliter wells (nanowells). Capture slides preincubated with mouse immunoglobulins were used for printing. Detection antibodies included fluorescence conjugated anti-IgG1, salivary gland lysates of C57BL/6 and SjS^s^ mice. Results indicate an increase in the frequency of IgG1-secreting cells in the spleen of SjS^s^ mice compared to C57BL/6 mice. Cells from the lymph node of SjS^s^ mice yield higher instances of IgG1 reactive against salivary gland antigens than cells from the lymph nodes of C57BL/6 mice. These data demonstrate the isotype-specific reactivity of antibodies during the autoimmune process, and further reveals significant differences in the non-autoimmune and autoimmune antibody repertoires. These results support the generation of self-reactive B cell repertoires during the autoimmune process, at the same time, verifying that microengraving of single cells might allow for identification of novel biomarkers in SjS.

## Introduction

Autoantibodies play a critical role in the pathogenesis and classification of autoimmune diseases. Although autoantibodies maintain particular specificity due to their antigen-binding motifs, their effector functions remain ambiguous. Autoantibodies known to be reactive against tissue and cell-specific antigens may or may not be associated with a particular disease etiology [1], [2]. For instance, the presence of circulating antibodies that block the nicotinic acetylcholine receptors at the postsynaptic neuromuscular junction is characteristic of myasthenia gravis [3], while antibodies against the muscarinic acetylcholine receptor type III (M3R) in Sjögren’s syndrome (SjS) are capable of impeding the neurotransmitters from binding the receptor for proper saliva stimulation [4]. Likewise, thyroid autoantibodies bind and stimulate the thyroid stimulating hormone receptor (TSHR), which causes hyperthyroidism in the autoimmune process of Grave’s disease [5], [6]. The challenge becomes apparent when attempting to classify autoantibodies that have no discernible pathogenicity in systemic autoimmunity. Autoantibodies identified in systemic lupus erythematosus (SLE) patients react against cardiolipin, fibronectin, golgin, histone H2A-H2B-DNA, and Ku-DNA-protein, however none have shown a clear etiological mechanism [7].

Whether it is an autoantibody-specific or autoantibody non-specific autoimmune diseases, one of the challenges is the sensitivity and feasibility of assays or techniques being used to enumerate such autoantibodies and the corresponding B cells. The standard laboratory methods for detection of these autoantibodies rely on many conventional techniques such as radial immunodiffusion assay (RID) or immunoprecipitation (IP). Recent advances in enzyme-linked immunosorbent assay (ELISA) or Luminex-based assays that use color-coded beads or microspheres conjugated with antigen of interest increases the efficiency of these assays by emphasizing high-throughput analyses for multiple antigens simultaneously. Two critical drawbacks of these methods are their lack of sensitivity and the need to use large quantities of serum extracted from patients to quantify detectable levels. Furthermore, the precise source of B cells producing these antibodies requires labor-intensive methodologies such as cloning or production of hybridomas. As a result, only the most prevalent antibodies can be measured. To circumvent these shortcomings, the application of microengraving appears to be beneficial [8]. Microengraving is a soft lithographic technique that uses a dense array of nanowells to print (identify) corresponding products secreted by individual cells confined in a subnanoliter well (nanowell) [8], [9]. A typical array comprises of 84,672 nanowells, each with a 50 µm×50 µm×50 µm dimension. Approximately a third to a half of the wells in the array contain one cell when plated with 500,000 cells in 300 µl volume [9]. As a result, ∼ 40,000 single cells can be analyzed at a time. In addition, single-cell resolution facilitates the measurement of antibodies secretion directly from the producing B cells at concentrations ranging from 0.1–1 µM [8], [9], [10].

Sjögren’s syndrome (SjS) is a human autoimmune disease characterized by loss of exocrine function as a result of chronic immune responses directed primarily against the salivary and lacrimal glands leading to xerostomia and xerophthalmia [11], [12]. SjS is a B cell-mediated autoimmune disease in which B cells and autoantibodies are suggested to play an important role in the exocrine glandular dysfunction [11], [13], [14]. Hyperproliferation and hyperactivity of autoreactive B cells frequently result in severe hypergammaglobulinemia in animal models and human patients with SjS [11], [15]. Furthermore, SjS patients, as well as animal models, develop specific autoantibodies against nuclear antigens, intracellular components, membrane proteins, and secreted products of exocrine tissues [16], [17], [18]. Between 40 and 70% of SjS patients’ sera contain autoantibodies that are reactive to SS-A/Ro and/or SS-B/La antigens [19], [20]. These two anti-nuclear autoantibodies (ANAs) are now used as diagnostic markers of SjS despite having an unknown role in the pathogenesis [21], [22], [23]. In contrast, recent studies have focused on anti-M3R autoantibodies, since preliminary data suggest that anti-M3R autoantibodies may be an important effector of glandular dysfunction by blocking parasympathetic neural transmission and internalization of receptor that desensitizes acinar cells to normal neural stimulations [4], [24].

The present study explores the isotype-specific autoantibody repertoires against salivary gland antigens by individual B cell isolated from SjS mice using microengraving. In addition, the study examines the reactive isotype-specific repertoires of B cells present in the secondary lymphoid organs specifically the spleen and cervical lymph nodes. Results indicated that there are significant numbers of IgG1-positive spleen cells of SjS mice compared to normal mice. Cells from the lymph nodes of SjS mice produce higher numbers of IgG1 autoantibodies reactive against self-antigens of the salivary glands compared to lymph nodes cells of normal mice. These data suggest that microengraving can be used to profile regional variations in the autoantibody repertoires and explore novel biomarkers in the disease.

## Materials and Methods

### Animals

C57BL/6 and C57BL/6.NOD-*Aec1Aec2* mice were bred and maintained under specific pathogen free conditions in the animal facility of Animal Care Services (ACS) at the University of Florida (Gainesville, FL, USA). Development of the C57BL/6.NOD-*Aec1Aec2* mouse and its SjS-like disease phenotype are described elsewhere [25]. Briefly, the C57BL/6.NOD-*Aec1Aec2* mouse was developed by introducing two genetic regions, one on chromosome 1 (designated *Aec2*) and the second on chromosome 3 (designated *Aec1*) derived from the NOD/LtJ mouse, into the SjS-non-susceptible C57BL/6J mouse. All animals were maintained on a 12 hr light-dark schedule and provided food and acidified water ad libitum. At times indicated in the article, mice were euthanized by cervical dislocation after deep anesthetization with isoflurane and their tissues freshly harvested for analyses. All experiments and analyses described in this article were performed using both male and female C57BL/6 (n = 4) and C57BL/6.NOD-*Aec1Aec2* (n = 4) mice, ranging from 20 to 24 weeks (wks) of age. The breeding and the use of animals as described in this study were approved by the University of Florida’s Institutional Animal Care.

### Isolation and Labeling of Salivary Glands Tissue Lysate

Salivary glands (SG) freshly explanted from C57BL/6.NOD-*Aec1Aec2* or C57BL/6J mice were lysed with Nonidet-P40 (NP40) buffer (150 mM sodium chloride, 1.0% NP-40, pH 8.0 50 mM Tris). Protein concentrations were determined by Bradford protein assay. To serve as fluorescent detection conjugates, SG proteins of C57BL/6.NOD-*Aec1Aec2* or C57BL/6J mice were labeled with Alexa Fluor 594 and Alexa Fluor 555, respectively according to manufacturer’s instructions (Thermo Scientific). In brief, 100 µg of proteins were incubated with Alexa Fluor dyes in presence of borate buffer for 3 hours (hrs) in the dark. Labeled proteins were mixed with suspended resin and loaded into a filter column. Following by a quick centrifugation, the final elute was collected and stored at −80°C for future use.

### Fabrication of Nanowells

Sylgard 184 silicone elastomer base (polydimethyl-siloxane, PDMS) and curing agent with 10∶1 weight ratio was combined and mixed vigorously. The mixture was degassed under vacuum for 2 hrs and poured into a custom-built aluminum mold containing silicon wafer with patterned arrays of posts (SU.8). The mixture was set to cure for 2 hrs at 80°C and adhered directly to a 3″×1″ in glass slide. The pattern on the master was transferred to the cured PDMS in bas-relief. In this experiment, a master was used that contained blocks of 7×7 nanowells, 4×4 blocks and 6 columns×18 rows with dimensions of 50 µm×50 µm×50 µm for a total of 84,672 nanowells per array [8].

### Preparation of Glass Slides for Microengraving

Microscopic glass slides (3″×1″) (Corning) were precleaned with 2.5 M NaOH in 57% ethanol for 2 hrs at room temperature (RT). Slides were rinsed with ultra pure water, followed by submerged in poly-L-lysine with gentle agitation for 30 min at RT. Poly-L-lysine-treated slides were dried by centrifugation at 500 rpm until dry. To ensure complete dryness, slides were placed in a vacuum oven for 5 min at 80°C.

Slides were stored for at least 14 days before coating with capture antibodies.

### Preparation and Imaging of Loaded Cells in Arrays of Nanowells

Spleens and cervical lymph nodes were freshly explanted, gently minced through stainless steel sieves, suspended in phosphate buffered saline (PBS) and centrifuged (1200 rpm for 5 minutes). Erythrocytes in spleens were lysed by 7 minute incubation in 0.84% NH_4_Cl. The resulting leukocyte suspensions were washed two times in PBS, counted and resuspended in culture media (RPMI 1640 medium, 10% FBS, 2 mM L-glutamine, 0.05 mM β-mercaptoethanol) at a density of 1×10^6^ cells/ml. A suspension of 1×10^6^ cells in 100 µl of culture media was stained with anti-CD19-Alexa Fluor 488, anti-CD4-Cy7 (Biolegend), and Calcein violet for live cell marker (Invitrogen). Stained cells were washed and suspended in 1 ml of media. 300 µl of solution containing 500,000 cells were loaded into an array of 50-µm nanowells. The cells were allowed to settle via gravity for 5 min. Excessive cells were rinsed off with media and a Lifterslip cover was placed on top to prevent evaporation from the nanowells. The arrays were imaged using an automated epifluorescence microscope equipped with a motorized stage and phase contrast, 405 nm, 488 nm and 647 nm wavelength filter sets.

### Microengraving

The array of nanowells was submerged in media to be rewetted after imaging. The array was gently placed in a chamber base of hybridization chamber (Agilent) and excess liquid was suctioned off using a glass pipette. The face of each treated dry glass slide as described previously [8] containing immunoglobulins (100 µg/ml goat anti-mouse IgG (H/L) (Zymed) and 100 µg/ml goat anti-mouse Ig (H/L) (Southern Biotechnology)) was placed on top of the array placed inside the chamber base. The assembly was secured by a finger-tightening screw and incubated at 37°C for 2 hrs. After incubation, the glass slide was carefully removed from the array and immediately placed in PBS. The nanowell array was quickly immersed in warm media for subsequent analysis if necessary.

### Analysis of Printed Microarrays

After microengraving, the glass slides were processed using Tecan HS Pro Hybridization system, as follows: 15 min hybridization with 3% nonfat milk in PBST (PBS with 0.5% Tween 20), washed twice with 1 min each, incubate for 45 min with IgG1-Alexa Fluor 488, B6 SG lysate labeled with Alexa Fluor 594 and Aec1Aec2 SG lysate labeled with Alexa Fluor 555 detection antibodies. The slide was vacuum dried and scanned using a Genepix 4200AL microarray scanner (Molecular Devices) with specific gain and power to maintain consistency among all the subsequent slides.

### Data Processing

Microarray micrographs and microscope images were processed to identify nanowells containing single cells with corresponding secretion of IgG1 antibody against SG proteins. In brief, Genepix Pro-6.1 software (Molecular Devices) was used to locate positive features on the scanned images of printed microarray using a custom GenePix Array List (GAL) designated with feature-indicators. Once all the features were found, each position in the array was analyzed to extract the mean-fluorescent intensities (MFI) for each channel corresponding to immunoglobulin. The data were extracted based on specific criteria for each channel. Initial screening included elimination of signal from cell debris, which is saturated; therefore percent of saturation (% sat) was selected with fluorescent (F) channel median vs. F channel % saturation of less than 2. Signal to noise ratio (SNR) of each channel was selected to be ≥1. This criterion ensures that signal is at least one standard deviation (STD) above the local background (B) intensity, increasing the likelihood that a true positive signal is chosen. A higher SNR is also suitable for eliminating false positives, but it can increase false negatives. To ensure that only data from wells with uniform signal were examined, %>Bchannel +2SD is selected at 50% or better with 100% being the best, set at 50%, anything below 50 was discarded. Similar to SNR, %>Bchannel +2SD ≥50% is equivalent to selecting for spots with SNR≥2. Coefficient of variation (CV) or signal uniformity is empirically set up at 100 to select uniform signals. Lastly, to compensate for any bleed through or possible cross-reactivity of the detection species, ratio of medians was applied. The channel of interest (i.e. 635) was positioned in the denominator and comparative channel (i.e. 555) was positioned in the numerator. Data point was set from 0.3–0.5. A value of 0.5 indicates the signal from the primary channel is at least twice a large as from the comparative channel. In this study, the ratio of medians was set at 0.3.

Analysis of the images of the cells recorded by the automated epifluoresence microscopy were inspected by using a custom software program to determine the number of cells present in each well and the MFI in each of the fluorescent channels. These data were matched with the corresponding antibodies detected by microengraving according to the unique location identification of each nanowell. This combined dataset was then filtered for analysis to include only wells occupied with single live cells.

### Statistical Analysis

Statistical evaluation was determined by using unpaired t test generated by the GraphPad InStat software (GraphPad Software Inc). The two-tailed p value <0.05 was considered significant.

## Results

### Microengraving

**Figure 1:**
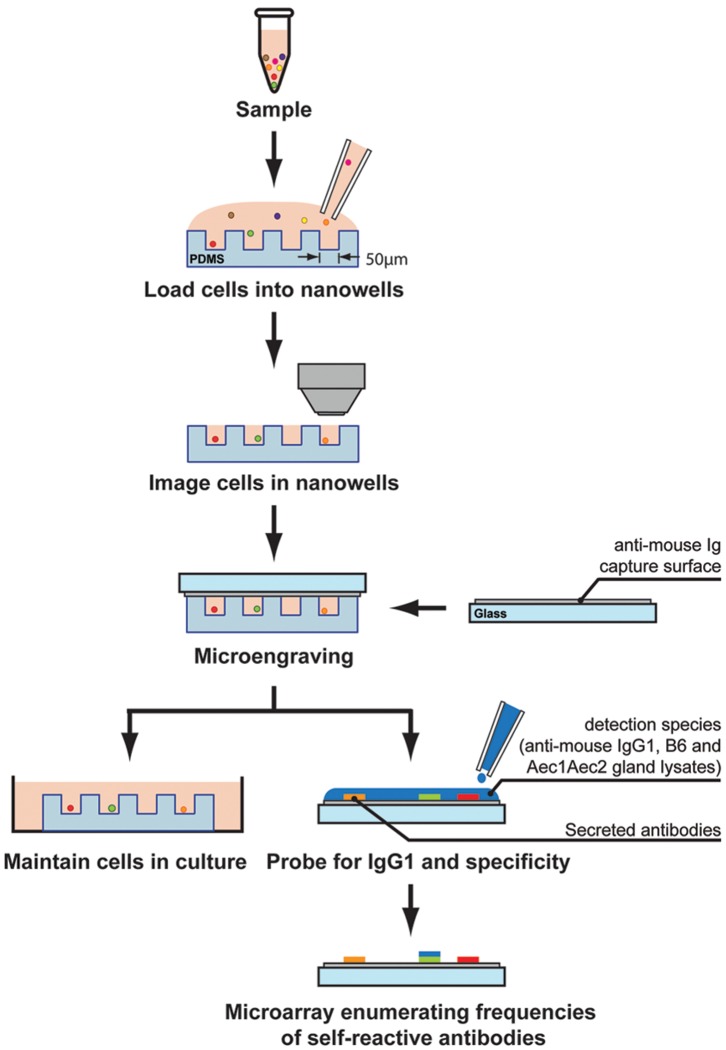
Illustration of microengraving. Arrays of nanowells with dimensions of 50 µm×50 µm×50 µm were used for microengraving. Spleen or cervical lymph nodes cells were loaded in the nanowells. Cells in the nanowells were imaged using an automated epifluorescence microscope. Micrograving is performed by hybridizing nanowells with capture slides containing anti-mouse Ig for 2 hrs at 37°C with 5% CO_2_. After incubation, nanowells containing intact live cells and capture slides were separated. A mixture of antibodies containing IgG1-Alexa Fluor 488, B6 SG lysate-Alexa Fluor 594 and *Aec1Aec2* SG lysate-Alexa Fluor 555 were added to the capture slides. Micrographs of microarrays were generating by scanning using a Genepix 4200AL microarray scanner.

Antibodies against Ro and La antigens are found in 60–90% and 30–60% of patients with primary SjS, respectively. However, patients with systemic lupus erythematosus (SLE) develop similar autoantibodies with lower frequencies (anti-Ro: 30–40% and anti-La: 10–15%) [19]. Anti-Ro and anti-La are being used as serological diagnostic biomarkers, however they provide neither specificity for the disease nor any clear etiology to the autoimmune process. One of the rationales for the complication is the laboratory test used to measure the levels of the antibodies. The primary serological test is ELISA, which favors the detection of only the most abundant antibodies. Furthermore, ELISA uses patients’ sera, which can degrade over time due to the nature of the antibodies and storage conditions. To circumvent these challenges and to further evaluate the source of the antibodies with multi-parametric analysis such as cell types, antibody isotypes and antigen specificity, the use of microengraving was tested. In the current study, lymphocytes from spleens or cervical lymph nodes isolated from normal C57BL/6 and SjS^S^ C57BL/6.NOD-*Aec1Aec2* mice were labeled with Calcein violet to mark live cells, CD19-FITC for B cells and CD4-Cy7 for mostly T cells. The labeled cells were dispersed and settled by gravity into the nanowells. The array of nanowells was imaged by automated high-speed epifluorescence microscopy to precisely locate specific nanowells with individual cells. Capture slides containing pan immunoglobulins (Ig) were used to hybridize the arrays containing cells [26]. Pan Ig enables the capture of most, if not all, the Ig isotypes secreted by B cells. To identify specific captured Ig, detection reagents including anti-IgG1-Alexa Fluor 488, C57BL/6 salivary glands lysate labeled with Alexa Fluor 594 and C57BL/6.NOD-*Aec1Aec2* salivary glands lysate labeled with Alexa Fluor 555 were used to determine the antigen specificity of B cells isolated from normal C57BL/6 and SjS^s^ C57BL/6.NOD-*Aec1Aec2* mice (**Figure 1** **)**. The integrated data indicate whether secreted IgG1 antibodies bind to C57BL/6 or C57BL/6.NOD-*Aec1Aec2* salivary gland proteins. In addition, features on the live cells in the nanowells can provide critical information on the individual cell type being examined.

**Figure 2:**
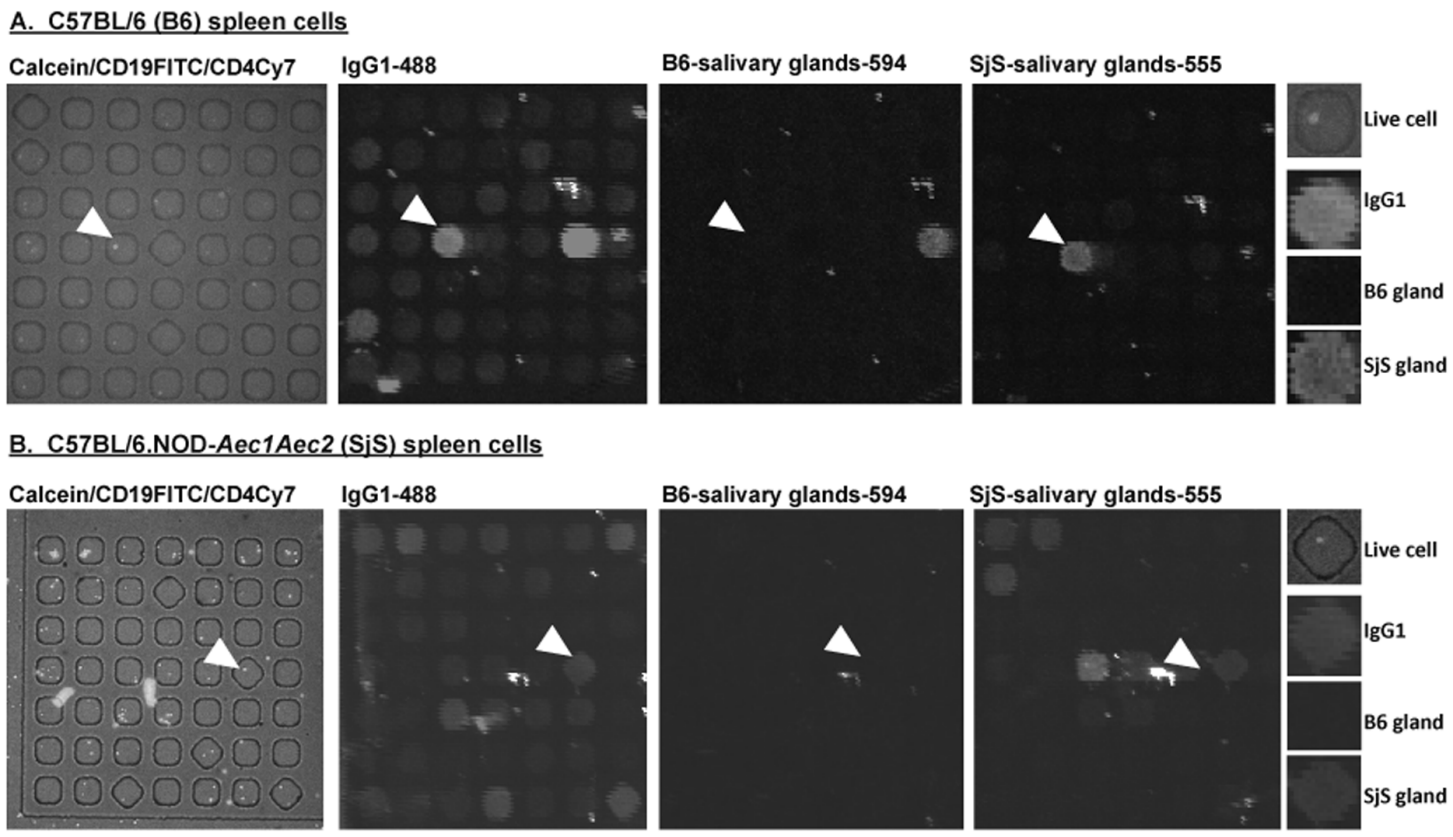
Characterization splenocytes from C57BL/6 and C57BL/6.NOD-*Aec1Aec2* mice using microengraving. **A.** Representative micrographs of live C57BL/6 splenocytes (n = 4) in nanowells labeled with Calcein (live cells), CD19-FITC and CD4-Cy7. Micrographs of matching microarray by microengraving showing detection signals for IgG1-Alexa Fluor 488, C57BL/6 (B6) salivary glands proteins labeled with Alexa Fluor 594 and C57BL/6.NOD-*Aec1Aec2* (SjS) salivary glands proteins labeled with Alexa Fluor 555. The last vertical panel illustrates the close-up features pointed by the arrows (Live cell: CD19FITC, IgG1: IgG1-488 signal, B6 gland: signal of antibody binding to salivary gland proteins isolated from B6 mice. SjS gland: signal of antibody binding to salivary proteins isolated from SjS mice. **B.** Representative micrographs of live C57BL/6.NOD-*Aec1Aec2* splenocytes (n = 4) in nanowells labeled with Calcein (live cells), CD19-FITC and CD4-Cy7. Micrographs of matching microarray showing detection signals for IgG1-Alexa Fluor 488, B6 salivary glands proteins labeled with Alexa Fluor 594 and SjS salivary glands proteins labeled with Alexa Fluor 555. The last vertical panel illustrates the close-up features pointed by the arrows (Live cell: CD19FITC, IgG1: IgG1-488 signal, B6 gland: signal of antibody binding to salivary gland proteins isolated from B6 mice. SjS gland: signal of antibody binding to salivary proteins isolated from SjS mice. All experiments were repeated at least twice for consistency.

**Figure 3:**
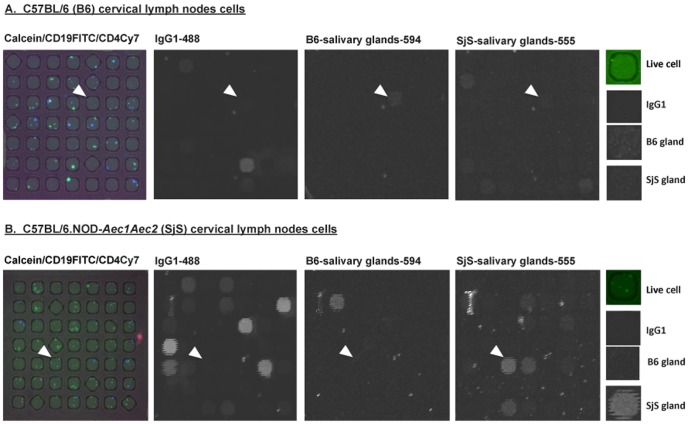
Characterization of cells from the cervical lymph nodes of C57BL/6 and C57BL/6.NOD-*Aec1Aec2* mice using microengraving. **A.** Representative micrographs of live cells from C57BL/6 cervical lymph nodes (n = 4) in nanowells labeled with Calcein (live cells), CD19-FITC and CD4-Cy7. Micrographs of matching microarray showing detection signals for IgG1-Alexa Fluor 488, C57BL/6 (B6) salivary glands proteins labeled with Alexa Fluor 594 and C57BL/6.NOD-*Aec1Aec2* (SjS) salivary glands proteins labeled with Alexa Fluor 555. The last vertical panel illustrates the close-up features (arrows) (Live cell: CD19FITC, IgG1: IgG1-488 signal, B6 gland: signal of antibody binding to salivary gland proteins isolated from B6 mice. SjS gland: signal of antibody binding to salivary proteins isolated from SjS mice. **B.** Representative micrographs of live cells from C57BL/6.NOD-*Aec1Aec2* cervical lymph nodes (n = 4) in nanowells labeled with Calcein (live cells), CD19-FITC and CD4-Cy7. Micrographs of matching microarray showing detection signals for IgG1-Alexa Fluor 488, B6 salivary glands proteins labeled with Alexa Fluor 594 and SjS salivary glands proteins labeled with Alexa Fluor 555. The last vertical panel illustrates the close-up features pointed by the arrows (Live cell: CD19FITC, IgG1: IgG1-488 signal, B6 gland: signal of antibody binding to salivary gland proteins isolated from B6 mice. SjS gland: signal of antibody binding to salivary proteins isolated from SjS mice. All experiments were repeated at least twice for consistency.

The application of microengraving to splenocytes of C57BL/6 or C57BL/6.NOD-*Aec1Aec2* mice (**Figure 2**) show a quantitative difference between binding of antigens from salivary glands of C57BL/6 or C57BL/6.NOD-*Aec1Aec2* mice. Live cells were visualized to confine in the nanowells of arrays with specific surface markers (CD19 B cells and CD4 T cells). The corresponding microarrays produced by microengraving show distinct signals for IgG1 antibody, as well as C57BL/6 or C57BL/6.NOD-*Aec1Aec2* salivary gland antigens. Representative examples of the data indicated by the arrowheads show that individual cells are positive for secreted IgG1 against C57BL/6.NOD-*Aec1Aec2* salivary gland proteins, but not C57BL/6 salivary gland proteins from splenocytes of either C57BL/6 or C57BL/6.NOD-*Aec1Aec2* mice (**Figures 2A & 2B**). Similar representative results were observed from cells isolated from the cervical lymph nodes of C57BL/6 and C57BL/6.NOD-*Aec1Aec2* mice (**Figure 3**). Interestingly, as indicated by the arrowheads, the cells are positive for IgG1 isotype against both C57BL/6 and C57BL/6.NOD-*Aec1Aec2* salivary gland proteins. These results indicate the versatility of using microengraving to profile the reactivity of individual B cells against targeted antigens of normal and autoimmune hosts. In addition, multi-parametric analysis can be achieved using microengraving to identify the individual types of cells, the isotypes of the antibodies, and the specificity for antigens in the glands.

### Frequency of IgG1 Isotype in the Spleens and Cervical Lymph Nodes

**Figure 4:**
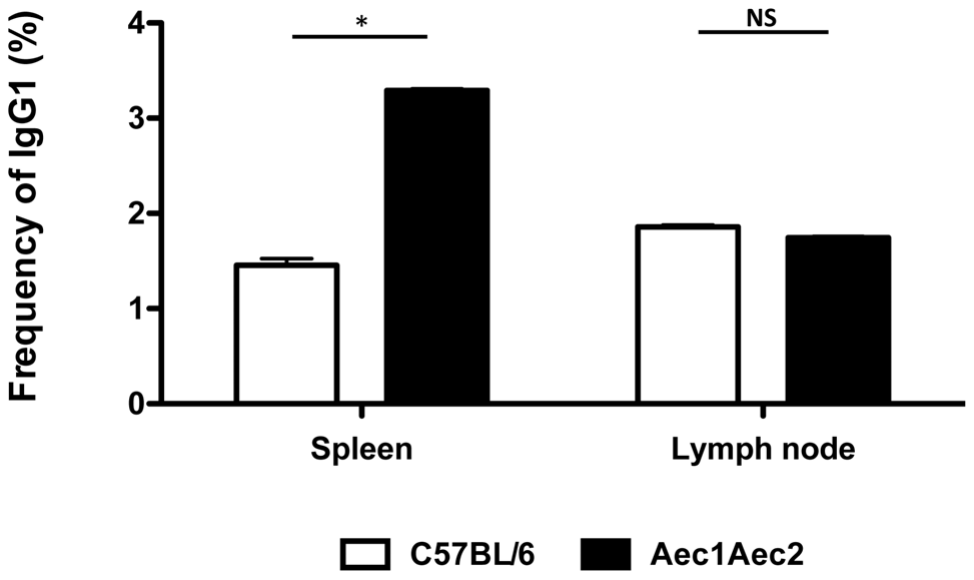
Frequency of IgG1. Enumeration of IgG1-secretion cells from arrays of nanowells occupied by single cells from the spleens and cervical lymph nodes of C57BL/6 (n = 4) and C57BL/6.NOD-*Aec1Aec2* mice (n = 4). Data extracted from the image processing using Genepix software were used to identify the appropriate signals. The data were correlated with the nanowell image data in which nanowells contained a single cell positive for both Calcein (live cells) and CD19. The frequency was determined by using the ratio of positive IgG1 signal from wells with single cells and the total number of wells with single cells. *p<0.05 by unpaired t test. NS: not significant.

IgG1 is the most abundant immunoglobulin found in the periphery. Previous results have shown that the levels of IgG1are highly elevated and predominantly expressed in SjS mouse models, specifically NOD and C57BL/6.NOD-*Aec1Aec2* mice compared to normal C57BL/6 mice (561.7±4.6 versus 347.7±5.4, respectively) [27]. The significantly high levels of IgG1 relative to with other isotypes contribute to the hypergammaglobulinemia commonly observed in SjS. Furthermore, our previous studies have shown that IgG1-isotypic autoantibodies against M3R were necessary and sufficient to block the receptor, resulting in the shutdown of saliva secretion, in contrast IgG2a-, IgG2b-, IgG3-, IgM-, and IgA-isotypic autoantibodies failed to elicit any direct effect on salivary function [4], [27], [28]. To identify IgG1-positive B cells and compare the frequency of these cells between C57BL/6 and C57BL/6.NOD-*Aec1Aec2* mice, nanowells containing only a single B cell were used for analysis. Splenocytes of C57BL/6.NOD-*Aec1Aec2* mice showed significantly higher numbers of IgG1-positive B cells (3.29±0.02) compared to splenocytes of C57BL/6 mice (1.46±0.07) (**Figure 4**), while cervical lymph nodes of both C57BL/6 and C57BL/6.NOD-*Aec1Aec2* mice had similar numbers of IgG1-positive B cells (1.86±0.02 and 1.74±0.01, respectively) (**Figure 4**). These results indicate that microengraving provides a highly efficient means to screen individual cells for a specific phenotype and/or function, even differentiating cell characteristics between normal and diseased individuals, such as non-SjS^s^ and SjS^s^ mice.

### Determination of IgG1-specific Antibodies against Salivary Gland Antigens

**Figure 5:**
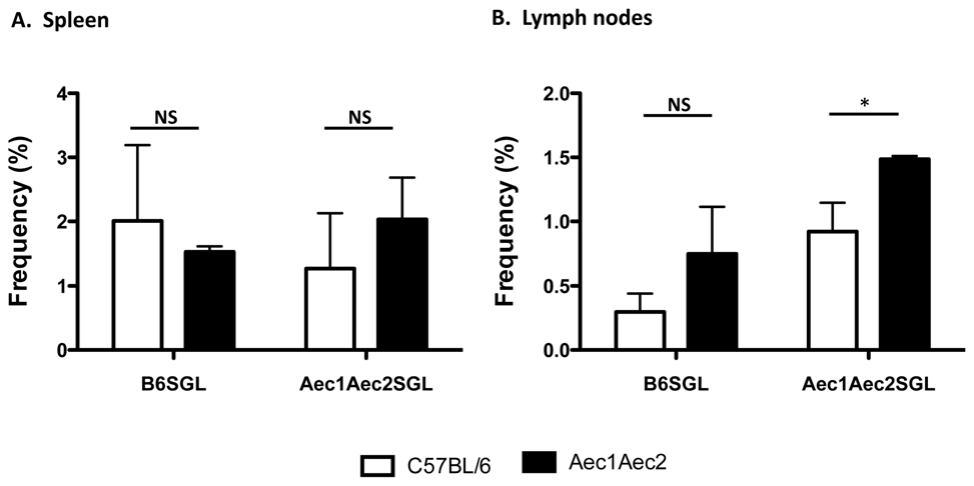
Frequency of IgG1-specific antibodies against autoantigens of salivary glands. Lymphocytes isolated from spleens (**A**) and cervical lymph nodes (**B**) of C57BL/6 (n = 4) and C57BL/6.NOD-*Aec1Aec2* (n = 4) mice were deposited onto the arrays of nanowells. Using data processing of micrographs of microarrays and epifluorescent images from the microscope, only signals that are originated from wells with single cells, positive for IgG1 and B6-salivary glands proteins or SjS-salivary glands proteins were used to analysis. *p<0.05 by unpaired t test. NS: not significant.

Analyses using microengraving can simultaneously identify each well containing single cells that produce a specific product, e.g., IgG1 antibody, and whether that product may differentiate between a normal and diseased state. We performed a direct comparison of IgG1-producing splenic B cells from C57BL/6 or C57BL/6.NOD-*Aec1Aec2* mice to determine if their IgG antibodies were reactive with salivary gland proteins. No differences were observed here (**Figure 5** **).** In a similar comparison of IgG1-producing B cells from lymph nodes, a significantly higher frequency of cells secreting IgG1 antibodies from SjS^s^ C57BL/6.NOD-*Aec1Aec2* mice were reactive against their own salivary gland proteins than those from C57BL/6 lymph nodes (1.49±0.03 versus 0.92±0.22, respectively, p<0.05). Although not statistically significant, lymph nodes cells of C57BL/6.NOD-*Aec1Aec2* mice appeared to also recognize more C57BL/6 salivary gland proteins (0.75±0.36 versus 0.30±0.14, respectively). Therefore, there is an intrinsic higher frequency of cells isolated from cervical lymph nodes of C57BL/6.NOD-*Aec1Aec2* mice that expressed IgG1 antibody reactive against their own salivary gland proteins relative to normal mice.

## Discussion

In the present study, we applied unconventional tools for single-cell analysis to identify individual IgG1-secreting B cells and enumerate the frequency of B cells secreting antibodies recognizing targeted self-salivary gland antigens in an animal model susceptible to the autoimmune disease (SjS). Results indicated that there are significant numbers of IgG1-positive spleen cells of SjS mice compared to normal C57BL/6 mice, however no difference in IgG1-positive cells was observed in the cervical lymph nodes. Interestingly, cells from the lymph nodes of C57BL/6.NOD-*Aec1Aec2* mice produce higher numbers of IgG1 autoantibodies reactive against self-antigens of the salivary glands compared to lymph nodes cells of C57BL/6 mice. No significant difference was observed among the fractions of B cells from the spleens of C57BL/6 and C57BL/6.NOD-*Aec1Aec2* mice reactive against self-antigens of the salivary glands. These data demonstrate that using arrays of nanowells and microengraving, secondary lymphoid organs such as spleen and cervical lymph nodes exhibited different self-reactive activities. More importantly, this animal model of SjS expressed higher isotype-specific and self-reactive or possibly autoimmune B cell repertoires, specifically IgG1 reactivity against salivary glands self-antigens than normal or non-autoimmune mice.

The humoral immune response is a complex process. The immune system is evolved by sustaining normal repertoires of autoantibodies functioning to maintain the immunological homeostasis [29], [30], [31]. These normal repertoires appear to not elicit any biological pathogenicity in a healthy or non-autoimmune background. In the general population, there are significant levels of autoantibodies present in sera of individuals, establishing a serological baseline for autoimmune prevalence. A recent study by Satoh et al. [32] using a cross-sectional analysis of 4754 individuals in the US revealed an ANA prevalence in 13.8% of US population ages 12 years and above. As anticipated, women had higher ANA prevalences than men (17.8% versus 9.6%) peaking at 40–49 years of age. Similar observations can also be found in the Japanese population, in which 26.0% of 2181 individual found to be positive for ANA with sera diluted at 1∶40 and 9.5% with sera dilution at 1∶160 with females exhibited higher prevalence in males [33]. At the time of these studies, none of the participants were clinically diagnosed with any autoimmune diseases. Such associations clearly demonstrate a naturally occurring repertoire of autoantibodies among the general population that does not associate or contribute to a particular autoimmune disease. These studies support our result that normal C57BL/6 mice have B cells in spleens and cervical lymph nodes that produce antibodies specific for antigens expressed in their salivary glands (**Figure 5**), but they do not present any detectable signs of autoimmunity. Interestingly, SjS mice appeared to show higher prevalence of B cells producing autoreactive antibodies in cervical lymph nodes cells but not splenocytes. Salivary glands and cervical lymph nodes are juxtaposed in the anterior vertebrate columns of the neck. Their interstitial connection has the potential to facilitate the migration of lymphocytes between the two organs. It remains elusive whether the autoreactive immune cells of the cervical lymph nodes or other organs contribute to sialadenitis. Based on the close proximity of the salivary glands to the cervical lymph nodes, it is likely that the lymphocytic infiltration might originate from the cervical lymph nodes.

This article is the first study that demonstrates B cells from cervical lymph nodes produce antibodies reactive against antigens of the salivary glands. It also suggests a potential evolution and influx of autoimmune cells from the regional salivary glands is important for tissue destruction. Further studies are underway to examine the clonal diversity of lymphocytes infiltrating the salivary glands, and which clonotypes are shared by the salivary glands and cervical lymph nodes.

Microengraving provides sensitivity at a single-cell resolution and multi-parametric analysis with high-throughput potential, essentially defining multiple factors contributing to the disease process. Our previous studies have shown that IgG1 antibody is directly involved in the secretory dysfunction of salivary glands [4], [28]. Using the NOD.B10-*H2^b^*.C-*Stat6* ^−/−^ and NOD.B10-*H2^b^*.C-*Il4* ^−/−^ mice, which fail to make IgG1 isotype antibody, we have determined that IgG1-isotypic autoantibodies against M3R were necessary and sufficient to block the receptor, resulting in the shutdown of saliva secretion [27], [28]. Both of these mouse lines have normal titers of IgG2a, IgG2b, IgG3, IgM, and IgA, yet failed to elicit any direct effect on salivary function. Our result has demonstrated that there is binding of antibodies to from the antigens of SjS gland, which lacks IgG1 signal. The data supports the observation that other isotypes might be reactive against antigens from the SjS gland, but their immuno-pathogenesis remains to be determined.

The current study establishes a strong foundation for how the application of microengraving can advance future studies to quantify and characterize unique IgG isotypes and autoantigens in autoimmune diseases for which autoantibodies play an important role in pathogenesis.

## References

[1] Martin F, Chan AC (2006) B cell immunobiology in disease: evolving concepts from the clinic. Annual review of immunology 24: 467–496. 10.1146/annurev.immunol.24.021605.090517 16551256

[2] Chan AC, Carter PJ (2010) Therapeutic antibodies for autoimmunity and inflammation. Nature reviews Immunology 10: 301–316. 10.1038/nri2761 20414204

[3] Dwyer DS, Bradley RJ, Urquhart CK, Kearney JF (1983) Naturally occurring anti-idiotypic antibodies in myasthenia gravis patients. Nature 301: 611–614. 6402708 10.1038/301611a0

[4] Cha S, Singson E, Cornelius J, Yagna JP, Knot HJ, et al (2006) Muscarinic acetylcholine type-3 receptor desensitization due to chronic exposure to Sjogren’s syndrome-associated autoantibodies. J Rheumatol 33: 296–306. 16465661

[5] Teng CS, Smith BR, Clayton B, Evered DC, Clark F, et al (1977) Thyroid-stimulating immunoglobulins in ophthalmic Graves’ disease. Clinical endocrinology 6: 207–211. 404101 10.1111/j.1365-2265.1977.tb03316.x

[6] Smith BR, Hall R (1974) Thyroid-stimulating immunoglobulins in Graves’ disease. Lancet 2: 427–431. 4137321 10.1016/s0140-6736(74)91815-7

[7] Lernmark A (2001) Autoimmune diseases: are markers ready for prediction? The Journal of clinical investigation 108: 1091–1096. 11602614 10.1172/JCI14234 PMC209538

[8] Ogunniyi AO, Story CM, Papa E, Guillen E, Love JC (2009) Screening individual hybridomas by microengraving to discover monoclonal antibodies. Nature protocols 4: 767–782. 19528952 10.1038/nprot.2009.40 PMC4034573

[9] Love JC, Ronan JL, Grotenbreg GM, van der Veen AG, Ploegh HL (2006) A microengraving method for rapid selection of single cells producing antigen-specific antibodies. Nature biotechnology 24: 703–707. 10.1038/nbt1210 16699501

[10] Story CM, Papa E, Hu CC, Ronan JL, Herlihy K, et al (2008) Profiling antibody responses by multiparametric analysis of primary B cells. Proceedings of the National Academy of Sciences of the United States of America 105: 17902–17907. 19004776 10.1073/pnas.0805470105 PMC2584674

[11] Nguyen CQ, Cha SR, Peck AB (2007) Sjögren’s syndrome (SjS)-like disease of mice: the importance of B lymphocytes and autoantibodies. Frontiers in Bioscience 12: 1767–1789. 17127420 10.2741/2187

[12] Nguyen CQ, Peck AB (2009) Unraveling the pathophysiology of Sjogren syndrome-associated dry eye disease. Ocul Surf 7: 11–27. 19214349 10.1016/s1542-0124(12)70289-6 PMC2861866

[13] Gordon TP, Bolstad AI, Rischmueller M, Jonsson R, Waterman SA (2001) Autoantibodies in primary Sjogren’s syndrome: new insights into mechanisms of autoantibody diversification and disease pathogenesis. Autoimmunity 34: 123–132. 11905842 10.3109/08916930109001960

[14] Jonsson R, Haga HJ, Gordon TP (2000) Current concepts on diagnosis, autoantibodies and therapy in Sjogren’s syndrome. Scand J Rheumatol 29: 341–348. 11132201 10.1080/030097400447525

[15] Sugai S, Masaki Y, Dong L (2004) Lymphoproliferative disorders in patients with Sjogren’s syndrome. Autoimmun Rev 3 Suppl 1S67–69. 15309805

[16] Lavoie TN, Lee BH, Nguyen CQ (2011) Current concepts: mouse models of Sjogren’s syndrome. Journal of biomedicine & biotechnology 2011: 549107. 21253584 10.1155/2011/549107 PMC3018660

[17] Fox RI, Kang HI (1992) Pathogenesis of Sjogren’s syndrome. Rheum Dis Clin North Am 18: 517–538. 1323135

[18] Hardin JA, Mimori T (1985) Autoantibodies to ribonucleoproteins. Clin Rheum Dis 11: 485–505. 2934209

[19] Harley JB, Alexander EL, Bias WB, Fox OF, Provost TT, et al (1986) Anti-Ro (SS-A) and anti-La (SS-B) in patients with Sjogren’s syndrome. Arthritis Rheum 29: 196–206. 3485431 10.1002/art.1780290207

[20] Routsias JG, Tzioufas AG (2007) Sjogren’s syndrome–study of autoantigens and autoantibodies. Clin Rev Allergy Immunol 32: 238–251. 17992591 10.1007/s12016-007-8003-8

[21] Vitali C, Bombardieri S, Jonsson R, Moutsopoulos HM, Alexander EL, et al (2002) Classification criteria for Sjogren’s syndrome: a revised version of the European criteria proposed by the American-European Consensus Group. Ann Rheum Dis 61: 554–558. 12006334 10.1136/ard.61.6.554 PMC1754137

[22] Novljan MP, Rozman B, Jerse M, Rotar Z, Vidmar G, et al (2006) Comparison of the different classification criteria sets for primary Sjogren’s syndrome. Scand J Rheumatol 35: 463–467. 17343255 10.1080/03009740600759860

[23] Shiboski SC, Shiboski CH, Criswell L, Baer A, Challacombe S, et al (2012) American College of Rheumatology classification criteria for Sjogren’s syndrome: a data-driven, expert consensus approach in the Sjogren’s International Collaborative Clinical Alliance cohort. Arthritis care & research 64: 475–487. 22563590 10.1002/acr.21591 PMC3349440

[24] Gao J, Cha S, Jonsson R, Opalko J, Peck AB (2004) Detection of anti-type 3 muscarinic acetylcholine receptor autoantibodies in the sera of Sjogren’s syndrome patients by use of a transfected cell line assay. Arthritis Rheum 50: 2615–2621. 15334476 10.1002/art.20371

[25] Cha S, Nagashima H, Brown VB, Peck AB, Humphreys-Beher MG (2002) Two NOD Idd-associated intervals contribute synergistically to the development of autoimmune exocrinopathy (Sjögren’s syndrome) on a healthy murine background. Arthritis Rheum 46: 1390–1398. 12115247 10.1002/art.10258

[26] Ronan JL, Story CM, Papa E, Love JC (2009) Optimization of the surfaces used to capture antibodies from single hybridomas reduces the time required for microengraving. Journal of immunological methods 340: 164–169. 19028499 10.1016/j.jim.2008.10.018

[27] Nguyen CQ, Gao JH, Kim H, Saban DR, Cornelius JG, et al (2007) IL-4-STAT6 signal transduction-dependent induction of the clinical phase of Sjogren’s syndrome-like disease of the nonobese diabetic mouse. J Immunol 179: 382–390. 17579059 10.4049/jimmunol.179.1.382 PMC2856075

[28] Gao J, Killedar S, Cornelius JG, Nguyen C, Cha S, et al (2006) Sjogren’s syndrome in the NOD mouse model is an interleukin-4 time-dependent, antibody isotype-specific autoimmune disease. J Autoimmun 26: 90–103. 16413168 10.1016/j.jaut.2005.11.004

[29] Hayakawa K, Asano M, Shinton SA, Gui M, Allman D, et al (1999) Positive selection of natural autoreactive B cells. Science 285: 113–116. 10390361 10.1126/science.285.5424.113

[30] Casola S, Otipoby KL, Alimzhanov M, Humme S, Uyttersprot N, et al (2004) B cell receptor signal strength determines B cell fate. Nat Immunol 5: 317–327. 14758357 10.1038/ni1036

[31] Gauld SB, Dal Porto JM, Cambier JC (2002) B cell antigen receptor signaling: roles in cell development and disease. Science 296: 1641–1642. 12040177 10.1126/science.1071546

[32] Satoh M, Chan EK, Ho LA, Rose KM, Parks CG, et al (2012) Prevalence and sociodemographic correlates of antinuclear antibodies in the United States. Arthritis and rheumatism 64: 2319–2327. 22237992 10.1002/art.34380 PMC3330150

[33] Hayashi N, Koshiba M, Nishimura K, Sugiyama D, Nakamura T, et al (2008) Prevalence of disease-specific antinuclear antibodies in general population: estimates from annual physical examinations of residents of a small town over a 5-year period. Modern rheumatology/the Japan Rheumatism Association 18: 153–160. 10.1007/s10165-008-0028-1 18283522

